# Statins exacerbate glucose intolerance and hyperglycemia in a high sucrose fed rodent model

**DOI:** 10.1038/s41598-019-45369-8

**Published:** 2019-06-19

**Authors:** Sriram Seshadri, Naimisha Rapaka, Bhumika Prajapati, Dipeeka Mandaliya, Sweta Patel, Christopher Shamir Muggalla, Bandish Kapadia, Phanithi Prakash Babu, Parimal Misra, Uday Saxena

**Affiliations:** 1 Institute of Science, Nirma University, Ahmedabad, India; 2 Dr. Reddy’s Institute of Life Sciences, University of Hyderabad Campus, Gachibowli, Hyderabad 500 046 India; 3 Department of Biotechnology & Bioinformatics, School of Life Sciences, University of Hyderabad, Gachibowli, Hyderabad 500 046 India; 4 Present Address: Marlene & Stewart Greene Baum Cancer Center, Department of Medicine, University of Maryland, Baltimore, Maryland United States of America

**Keywords:** Insulin signalling, Pharmacodynamics

## Abstract

Statins are first-line therapy drugs for cholesterol lowering. While they are highly effective at lowering cholesterol, they have propensity to induce hyperglycemia in patients. Only limited studies have been reported which studied the impact of statins on (a) whether they can worsen glucose tolerance in a high sucrose fed animal model and (b) if so, what could be the molecular mechanism. We designed studies using high sucrose fed animals to explore the above questions. The high sucrose fed animals were treated with atorvastatin and simvastatin, the two most prescribed statins. We examined the effects of statins on hyperglycemia, glucose tolerance, fatty acid accumulation and insulin signaling. We found that chronic treatment with atorvastatin made the animals hyperglycemic and glucose intolerant in comparison with diet alone. Treatment with both statins lead to fatty acid accumulation and inhibition of insulin signaling in the muscle tissue at multiple points in the pathway.

## Introduction

Statins are the best-known cholesterol lowering drugs currently used in clinical practice. They inhibit the rate limiting enzyme for cellular cholesterol biosynthesis, HMG CoA reductase and deprive the cells of cholesterol^1–5^. While they are considered lifesaving in the treatment of cardiovascular disease, after their use in millions of patients it is now been uncovered that they have a side effect related to diabetes^6–13^. Data from retrospective clinical studies suggest that statins increase the probability of inducing type 2 diabetes^14–21^. As a result of this there is now a black box warning by the USFDA to use caution in their use in patients^22^.

While the human data on statins and propensity for causing type 2 diabetes is enticing, it is difficult to attribute the effect to statins alone because the patients may have several variables such as use of other drugs, other risk factors like obesity, family history, age, ethnicity etc.^23–27^. Although the data are controlled for these variables, it is challenging to conclusively ascribe these effects to statins. In addition, a more pertinent question from a clinical stand point is what impact would statins have in pre-diabetic conditions, a very likely target population for statin treatment since most pre-diabetics are also dyslipidemia^28–30^. To address these questions, we designed the studies presented here. In an animal model, we can study the impact of statins in a controlled setting avoiding variables such as those seen in humans^23–27^. In addition, it is possible to directly asses the mechanism of how statins may cause glucose intolerance and hyperglycemia in an animal model.

In the current *in vivo* studies, we had two objectives in mind

- Can statins exacerbate glucose intolerance in high sucrose fed animal models?
- What could be the potential mechanism of this effect?

**Figure 1:**
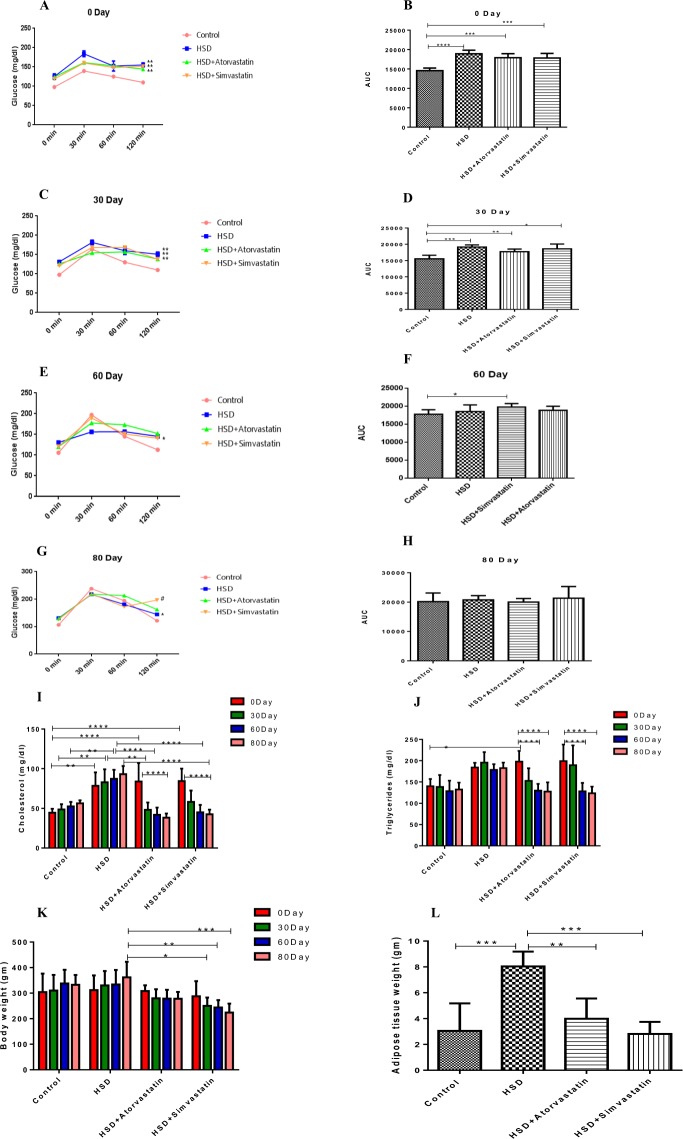
Effect of low dose Atorvastatin (10 mg/kg/day) or Simvastatin (20 mg/kg/day) treatment up to 80 days on different metabolic parameters in HSD induced pre-diabetic rats. (**A**) OGTT of diabetic animal (at day 0 of statin induction); (**B**) AUC_glucose_ for the 0 day OGTT; (**C**) OGTT on 30^th^ day; (**D**) AUC_glucose_ of 30^th^ day OGTT; (**E**) OGTT on 60^th^ day; (**F**) AUC_glucose_ of 60^th^ day OGTT; (**G**) OGTT of 80^th^ day; (**H**) AUC_glucose_ of 80^th^ Day OGTT; (**I**) Comparison of Cholesterol of animal from 0 to 80^th^ day; (**J**) Comparison of triglyceride of animal from 0 to 80^th^ day; (**K**) Comparison of body weight of animal from 0 to 80^th^ day; (**L**) Weight of Adipose tissue at end of study (after 80 days of induction. Significance (p value) calculated using Graph Pad prism V6.01 and one-way ANOVA performed, where, *p < 0.05, **p < 0.01, ***p < 0.001 and ****p < 0.0001.

We used high sucrose diet (HSD) fed rats as the model in our studies. In this model, the fasting plasma glucose levels are in the range of 100–120 mg/ml (Fig. 1A) along with an increased plasma cholesterol in the range of 78–90 mg/dl (Fig. 1I) and triglycerides in the range of 160–180 mg/dl (Fig. 1J) levels, similar to pre-diabetic humans. Therefore, we explored the effect of statins in this model. We utilized two statins: atorvastatin and simvastatin, the most prescribed statins to delineate potential role in glucose intolerance.

In our previous study using L6 myotubes *in vitro* model, we reported that statins caused intracellular accumulation of free fatty acids which in turn inhibited insulin signaling pathway at multiple points^31^. So, in this current study, we also explored whether such pathways are applicable *in vivo*.

Finally, there have been limited published studies that addressed the role of statins to cause glucose intolerance and hyperglycemia *in vivo* with a view of trying to understand what could happen in a human condition such as pre-diabetes^32–35^.

## Results

### Statins promote hyperglycemia and glucose intolerance in HSD rats

To explore whether statins can increase hyperglycemia, the following experimental paradigm was used. We induced hyperglycemia in animals by putting them on a HSD for 90 days. Post induction, we treated HSD fed rats orally with low dose atorvastatin (10 mg/kg/day) and low dose simvastatin (20 mg/kg/day) for up to 80 days on daily basis and compared various metabolic parameters. Four animal groups were compared, a) control, chow fed animals, b) HSD alone group, c) HSD plus atorvastatin treatment and d) HSD plus simvastatin treatment.

The average fasting blood glucose levels on day 0, i.e the day of statin treatment initiation in HSD animals was 125 mg/dl versus the levels in chow fed animals was 98 mg/dl (*p* = 0.02). As shown in Fig. 1A,B, oral glucose tolerance tests (OGTT) showed that at day zero (before initiation of statin treatments), there was no statistically significant difference in glucose disposal curves amongst the three HSD groups. As expected, the HSD groups had significantly different glucose disposal groups compared to control chow fed animals. This demonstrates that HSD fed animals were hyperglycemic.

After treatment with low dose statins, the fasting glucose levels in these animals were increased (Fig. 1C,E,G) suggesting that the statin treatment promotes hyperglycemia over and above HSD. However, there was no statistically significant difference in glucose disposal curves amongst the three HSD groups (Fig. 1D,F,H at days 30, 60 and 80 days of statin treatment) although there was a trend towards worsening of glucose tolerance by statins especially at 120 min after oral glucose dosing.

We also examined the effect of low dose statin treatment on cholesterol and triglyceride levels at day 80 of statin treatment. When compared with HSD fed animals, there was 58.8% (*p* < *0*.*0001*) and 54.2% *(p* < *0*.*0001)* statistically significant decrease in cholesterol levels when treated with atorvastatin and simvastatin respectively. Similarly, we observed 30% (*p* < *0*.*001*) decrease in triglyceride levels with both statin treatment. As expected, both atorvastatin and simvastatin had profound lowering of both plasma cholesterol and triglyceride (Fig. 1I,J). This shows that the statins were effective in lowering lipids in this model and were efficacious as expected.

We measured the total body and adipose tissue weight. In this model, because of the HSD there is clear weight gain in the untreated HSD group maintained on the diet (Fig. 1K,L). Both atorvastatin and simvastatin decreased the body weight gain by 23% and 38% (*p* < 0.001) as well as adipose tissue weight statistically significantly by 50% (*p* < 0.01) and 65% (*p* < 0.001) respectively compared HSD animals alone possibly due to correction in lipid profile especially triglycerides which can provide free fatty acids for fat accumulation in the adipose or through other unknown mechanisms (Fig. 1I,J).

### Statins induce fatty acid accumulation and gene expression changes in muscle

We then designed a 30-day statin treatment paradigm in HSD animals to better understand the fatty acid levels and gene expression changes in the muscle, a major target organ for glucose clearance under insulin stimuli. Here, we treated HSD fed rats with high dose statins using atorvastatin (20 mg/kg) and simvastatin (30 mg/kg) for a shorter duration (30 days) to push hard on the prospect of statins worsening hyperglycemia and insulin signaling. The three HSD groups were randomized and assigned to HSD alone, HSD plus atorvastatin treated and HSD plus simvastatin treatment.

**Figure 2:**
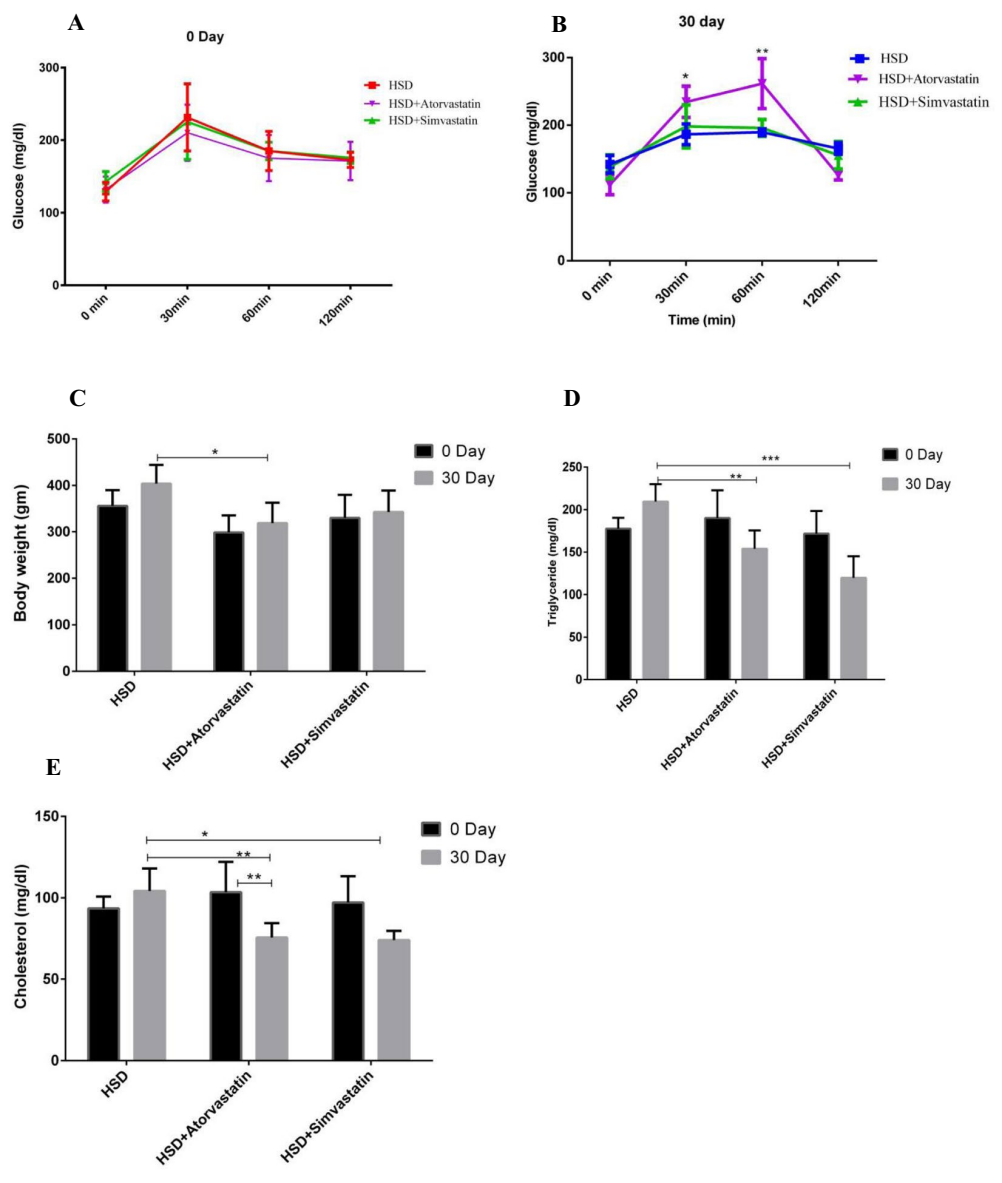
Effect of high dose Atorvastatin (20 mg/kg/day) or Simvastatin (30 mg/kg/day) treatment for 30 days on different metabolic parameters in HSD induced pre-diabetic rats. (**A**) OGTT in HSD animals in all groups (at day 0 of statin treatment); (**B**) OGTT in animals after 30 days. (**C**) Comparative graphs for body weights; (**D**) Comparison of Triglyceride levels; (**E**) Comparative graph for the Cholesterol levels. Significance (p value) calculated using Graph Pad prism V6.01 one-way ANOVA, *p < 0.05, **p < 0.01, ***p < 0.001, ****p < 0.0001.

Using the high dose statin treatment regimen, firstly, we found that OGTT disposal curves at day zero, the curves between the three animal groups were not different (Fig. 2A). However, at day 30 after statin treatment, there were differences with worsening of glucose disposal in statin treated groups especially at 30 and 60 minutes in OGTT. There was statistically significant effect of atorvastatin (*p* = 0.008) at these time points, but simvastatin showed trend towards worsening of glucose disposal (Fig. 2B). We also found that atorvastatin and simvastatin reduced body weight by 21% (*p* < 0.05) and 15% respectively. Further atorvastatin and simvastatin decreased plasma cholesterol significantly by 27% (*p* < 0.01) and 29% (*p* < 0.05) and triglyceride levels by 26% (*p* < 0.01) and 42% (*p* < 0.001) respectively (Fig. 2C–E).

**Figure 3:**
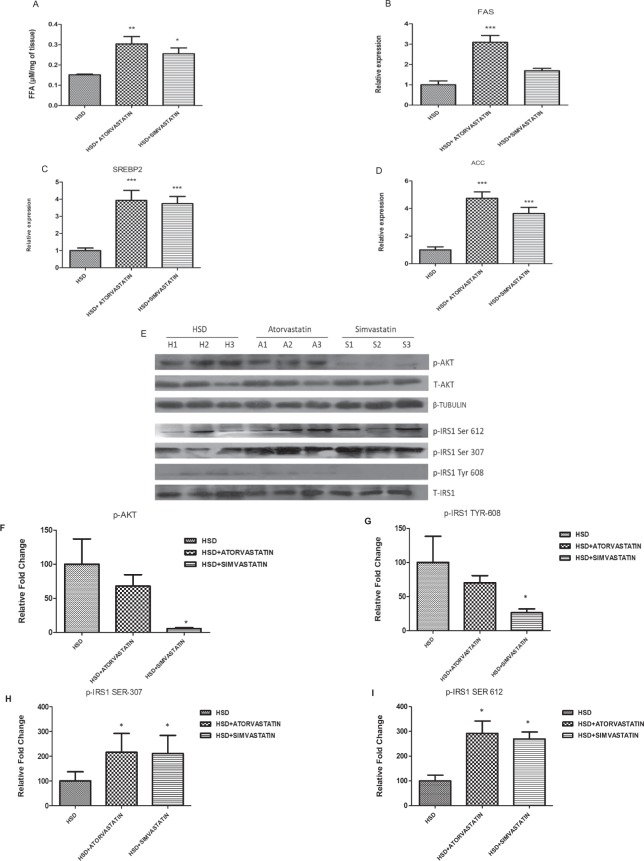
Effect of statins on the lipid and gene expression profiles in skeletal muscle of HSD rats. (**A**) Simvastatin or Atorvastatin treatment enhanced free fatty acid levels in skeletal muscle tissue of HSD animals. HSD animals were treated orally with Atorvastatin (20 mg/kg/day) and Simvastatin (30 mg/kg/day) for 30 days. Animals were euthanized, skeletal muscle tissues were isolated and free fatty acid levels were measured. **(B**–**D)** Simvastatin or Atorvastatin treatment enhanced FAS, SREBP2 and ACC1 gene expression in muscle tissue of statins treated HSD animals**:** mRNA expression of rattus FAS, SREBP2 and ACC1 in statin muscle tissue of HSD animals. *p < 0.05, **p < 0.01, ***p < 0.001 versus corresponding muscles of control HSD animals. **(E)** Simvastatin or Atorvastatin treatment inhibited insulin signalling cascade: Skeletal Muscles tissues were collected after statin treatment of HSD animals and probed by western blots for phosphorylation status of IRS-1 (ser^307^and ser^612^, tyr^608^) and pAKT. Total IRS-1, total AKT and β-actin served as loading control. The cropped blots were run under the same experimental conditions. The full-length blots are included in Supplemental Fig. 1(Fig. S1). (**F–I**) Densitometric quantification results of western blots from (**E**). Values are shown as mean ± SD after normalizing with the corresponding protein content and expressed relative to basal (total AKT and β-tubulin) for pAKT and relative to basal (total IRS-1) for p-ser^307^, p-ser^612^ and p-tyr^608^ of muscle tissues of control HSD animals which was set to 1 versus muscle tissues of statin treated HSD animals.

We next examined the impact of high dose statin treatment on muscle fatty acid content. We previously reported that simvastatin treatment in rat L6 myotubes hampers insulin induced glucose uptake due to accumulation of free fatty acids, a potent inhibitor of insulin signaling^31^. Supporting our previous observations, we found that atorvastatin and simvastatin treatment led to significantly higher amounts of fatty acid content in the muscle by 100% (*p* < 0.01) and 68% (*p* < 0.05) respectively relative to HSD treatment alone (Fig. 3A).

To further substantiate our observations, we determined the expression of genes associated with fatty acid synthesis. The gene expression of FAS (fatty acid synthase) increased by 209% (*p* < 0.001) and 69%, SREBP2 (sterol regulatory element-binding protein 2) increased by 292% (*p* < 0.001) and 274% (*p* < 0.001) and ACC (acetyl coA carboxylase) increased by 374% (*p* < 0.001) and 263% (*p* < 0.001) in the muscle of atorvastatin and simvastatin treated HSD rats respectively (Fig. 3B–D). In all of the above studies including fatty acid accumulation, the effect of atorvastatin was generally superior to simvastatin perhaps due to which atorvastatin impact on glucose disposal was significant while simvastatin only showed a trend towards worsening.

### Statins impair insulin signaling pathway at multiple steps

We then explored the impact of statins on insulin signaling cascade, the primary signaling responsive for clearance of glucose from circulation (Figs. 3E and S1). First, we explored the impact of statin on IRS1 (Insulin receptor substrate1) phosphorylation in the muscles of the rats. As shown in Fig. 3F, phosphorylation status of Akt, secondary messenger of insulin signaling cascade, was reduced by 32% and 94% (*p* < 0.05) upon atorvastatin and simvastatin treatment respectively compared to HSD rats alone. We noted that tyrosine ^608^ phosphorylation of IRS (an efficient marker to quantify the impact of insulin signaling cascade) (*p* < 0.05) was hardly detected (Fig. 3G) whereas inhibitory phosphorylation of IRS at ser^307^ was enhanced by 115% (*p* < 0.05) and 111% (*p* < 0.05) and at ser^612^ was enhanced by 191% (*p* < 0.05) and 169% (*p* < 0.05) with atorvastatin and simvastatin treatment respectively compared to HSD rats alone (Fig. 3H,I). Collectively, these data suggest that statins potentiate hyperglycemia and glucose intolerance under pre-diabetic condition via specific insulin-specific molecular defects in skeletal muscles.

## Discussion

The studies presented here suggest that statins may promote glucose intolerance and hyperglycemia in an HSD fed animal model reminiscent of human pre-diabetes. The number of pre-diabetics is growing worldwide at an alarming rate and many of them will convert into full blown diabetics. Most pre-diabetics also present themselves with the co-morbidity of dyslipidemia and are often prescribed statins. One interesting point to consider is that we have tried to mimic the effects of statin therapy in our acute animal studies, but in the clinical setting where statins are used chronically, the effects may be exacerbated even further.

In our findings, atorvastatin appears to be detrimental while simvastatin was not impactful on inducing glucose intolerance. One straight forward explanation could be that atorvastatin is a more potent statin relative to simvastatin, but we cannot exclude other drug specific effects or due to differences in the bioavailability of the two drugs in this sucrose fed model.

Another important finding of the current work delves into the mechanism of how statins may cause glucose intolerance. A thorough understanding of the mechanism of statin attenuating insulin signaling cascade will aid in designing counter measure to control this undesirable side effect in the future. Exposure to statin significantly enhanced intracellular free fatty acid levels in skeletal muscle which may inhibit insulin signaling cascade to hamper glucose clearance. Free fatty acids are known to inhibit insulin signaling via the PKC pathway^36–38^. Cumulatively, these data suggest that *in vivo* statins may induce glucose intolerance (in part) by free fatty acid mediated pathway although other unknown mechanisms cannot be ruled out.

A caveat in our interpretations of how statins may cause glucose intolerance is that it is focused on muscle tissue. It has also been shown by others that fluvastatin, another member of statin family of drugs, regulates insulin sensitivity in adipose tissue^32^.

We propose that high doses of atorvastatin but not simvastatin may exacerbate the hyperglycaemic effect of high sucrose feeding on glucose tolerance and that both statins seem to promote fatty acid accumulation and down regulation of basal insulin signalling molecules in muscle tissue.

### Methodology

#### Drugs

Atorvastatin and simvastatin are gifts from Dr. Reddy’s Laboratories Limited, Hyderabad, Telangana, India. Simvastatin and atorvastatin were 99.30% and 100% pure respectively as per the purity certificates provided by the manufacture (Figs S2A and S3A). All experiments were conducted with these highly pure drugs. To ascertain any degradation of the compounds upon storage we checked the purity of the both the statins by chromatographic HPLC methods. Purity of simvastatin and atorvastatin were 99.30% and 97.11% respectively (Figs S2B and S3B). The purity data show that even after storage there is no degradation of simvastatin and very little degradation of atorvastatin. The purity data and the ability of these drugs to lower cholesterol in our *in vivo* studies indicate that the statins performed as expected.

### Methodology of the determination of the chromatographic purity of statins by HPLC

#### Column

X- Bridge C_18_ 150 × 4.6 mm, 5.0 µm; *Mobile Phase A*: 5 mM Ammonium acetate in water; *Mobile Phase B*: Acetonitrile; *Elution type*: Gradient; *Gradient program*: Time (minutes), % A and % B: [0.01 min, 90% A: 10% B; 23.0 mins, 10% A: 90%B; 30.0 mins, 10% A: 90% B; 31.0 mins, 90% A: 10% B and 35 mins, 90% A: 10% B]; *Column temperature*: 30 °C; *Flow rate*: 1.0 ml/min; *Injection volume*; 5.0 µl; *Run time*: 35 mins; *Wavelength*: 245 nm; *Diluent*: DMSO and *sample preparation*; 0.5 mg/ml.

#### Animal treatment

All the methods were carried out in accordance with the approved guidelines. Experimental protocol involving animals was reviewed and approved by the Animal Ethical Committee of Institute of Science, Nirma University, Ahmedabad, India (Protocol No. IS/BT/FAC-13-1009). In the present study, inbred healthy adult Wistar albino rats, weighing around 150 ± 10 g were maintained in polypropylene cages under standard photoperiod and temperature controlled rooms. During the quarantine period, all the animals were fed with standard chow diet and water was provided *ad libitum*.

For low doses of statin study (Fig. 1), 25 animals were randomly segregated and fed with standard chow diet fed (control group i.e. 5 animals) or high sucrose diet (HSD, 65% sucrose, treated group; 20 animals) for a period of 90 days^39^. The HSD was prepared in house following the preparation formula mentioned in^40^. Following 90 days of HSD feeding, 15 animals were further regrouped in three groups with 5 animals each. One group was maintained on HSD while another two groups were treated with 10 mg/kg/day atorvastatin and 20 mg/kg/day simvastatin orally for 80 days along with HSD.

For high doses of statin study (Fig. 2), twenty animals were fed HSD for a period of 90 days. Following 90 days of HSD induction, 15 animals were further regrouped in three groups with 5 animals each. One group was fed HSD alone while another two groups were treated with 20 mg/kg/day atorvastatin and 30 mg/kg/day simvastatin orally for 30 days along with HSD.

All animal groups were euthanized upon completion of statin treatment periods of 80 days and 30 days, respectively (Figs 1 and 2). Blood was collected into vials with and without anticoagulant by puncturing retro-orbital plexus then further centrifuged (10,000 rpm, 10 min) to obtain plasma or serum samples, respectively. The samples were frozen (−20 °C) and stored for further blood biochemical analysis like fasting blood sugar, total cholesterol and triglyceride using Accucare diagnostic kit following as per the protocol mentioned by manufacturer.

All the 15 animals were bled thrice with an interval of 24 hours prior to their autopsy upon completion of their individual groups dose administration i.e., HSD induction and statin treatment.

#### OGTT

Oral glucose tolerance test (OGTT) was performed on overnight fasted rats on specific days as mentioned (Figs. 1 and 2). Briefly, after fasting, animals were given a glucose load (2 g/kg) orally. Blood was collected at regular intervals of 0 mins, 30 mins, 60 mins and 120 mins. Glucose concentrations were determined with a Free Style Optium H Blood Glucose Monitor (Abbott, Maidenhead, UK). Area under the curve for glucose (AUC_glucose_) was calculated using the trapezoidal rule^40^.

#### Estimation of cholesterol

1 ml of reagent has been added to 10 μl of the prepared serum (Cholesterol based on CHOD/POD - a kit supplied Lab-Care Diagnostics (India) Pvt. Ltd). The solution is allowed to mix well and incubated at 37 °C for 5 minutes. The solution turns to pinkish red color. The free cholesterol was oxidized by cholesterol oxidase (CO) to produce cholesten-3-one with the simultaneous production of hydrogen peroxide, which oxidatively couples with 4-aminoantipyrine and phenol in the presence of peroxidase (POD) to yield Quinoneimine dye with maximum absorption at 505 nm.

#### Estimation of Triglycerides

1 ml of reagent has been added to 10 μl of the prepared serum (Triglycerides based on GPO/POD - a kit supplied by Lab-Care Diagnostics (India) Pvt. Ltd). The solution is allowed to mix well and incubated at 37 °C for 5 minutes. The solution turns to brownish red color. Triglycerides were hydrolyzed by lipase to glycerol and free fatty acids. Glycerol is phosphorylated by ATP in the presence of glycerolkinase (GK) to Glycerol-3-Phosphate (G-3-P) which is oxidized by the enzyme glycerol-3-Phosphate oxidase (G-P-O) producing hydrogen peroxide. Hydrogen peroxide so formed reacts with 4-aminoantipyrine and 4-Chlorophenol in the presence of enzyme peroxidase (POD) to produce Quinoneimine dye compound which is then read at 505 nm.

### Western blots analysis

Skeletal muscle was homogenized in RIPA buffer (Glycerol-10%, 150 mM NaCl, 50 mM Tris-HCl (pH-7.4), 12 mM Sodium deoxy cholate, 1% NP4O/Triton X-100, 2.5 mM EDTA and 1.8 mM SDS) and centrifuged for 30 min at 13000 g. The extracted protein was estimated and 75 µg of total protein lysates were loaded on 10% SDS acrylamide gel and subjected to electrophoresis. Subsequently, the proteins were transferred to PVDF membrane (Millipore, MA, USA). The membranes were blocked with 5% non-fat dry milk in TBST buffer TBS; 10 mM TRIS (pH 8.0), 150 mM NaCl) and probed for T-Akt (1:1000, CST, Cat.no: 9272), p-Akt (1:1000, CST Cat.no:9271), T-IRS1(1:1000, Merck Millipore, Cat.no: 06-248), p-IRS1 ser ^307^ (1:1000, Merck Millipore, Cat.no: 05-1087, phospho-IRS1 ser ^612^(m)/ser ^616^(h) (1:1000, Merck Millipore, Cat.no: 09-448), phospho-IRS1 tyr ^612^(h)/tyr ^608^(m) (1:1000, Abcam, Cat.no: ab4868) and β-Tubulin (1:1000, Santa curz, Cat.no: SC-5274). Chemiluminescence of T-Akt, p-Akt, β-Tubulin were determined by using ECL reagent on X-ray films and that of T-IRS1, p-IRS1 ser ^307^, P-IRS1 ser ^612^, P-IRS1 tyr ^608^ were developed using Chemidoc touch (Biorad 1708370). Tubulin, T-Akt and p-Akt bands are from the same gel whereas bands of T-IRS1, p- IRS1 ser ^307^, p-IRS1 ser ^612^ and p-IRS1tyr ^608^ are from another gel. Experiments were repeated 3 times (n = 3) and a representative gel is shown.

### Real-time PCR

100 mg rat skeletal muscle tissue was homogenized using RNA Isoplus reagent (Takara, Cat.no: 9108) and chloroform was added to the homogenate. After phase separation, the top layer containing RNA was collected and RNA is precipitated by isopropanol extraction step. cDNA synthesis was performed by taking 5 µg total RNA. qPCR was performed by using the following oligos for FAS (Forward Primer: **GCTTCGCCAACTCTACCATG**; Reverse Primer: **AGATAATGCCCACGTCACCA**), for SREBP2 (Forward Primer: **TGCCTCACTCTCTGGAAAGG**; Reverse primer: **GTAGGCCGCTGACATTGAG**), for ACC (Forward Primer: **AGTCCATGTCCACTCAAGCA**; Reverse Primer: **TGCCAATCTCGTTTCCTCCT**) using ABS, Step- one plus master cycler (Invitrogen, CA, USA). mRNA expression was normalized to 18 s RNA as reference gene. The experiments were performed in duplicates and repeated three times (n = 3).

### Free fatty acid quantification

Free Fatty acid levels were quantified by using FFA quantification kit (Sigma, cat no: MAK044). 50 mg rat skeletal muscle tissue was homogenized in 1% Triton X-100 in chloroform (w/v). The samples were centrifuged at 13000 g for 10 min. The organic phase was allowed to air dry at 50 °C. The dried lipids were resuspended in the fatty acid buffer and FFA was estimated as per the manufacturer’s instructions. The values were reported as µM/mg of tissue^41^. The experiments were performed in duplicates and repeated three times (n = 3).

### Statistical analysis

Data is expressed as mean ± standard deviation. For comparison between 2 or more groups one-way ANOVA is used followed by either Dunnett’s post hoc analysis (for comparison to one control) or Bonferroni’s multiple comparison test (for comparing two or more sample set), *p* < 0.05 is considered as significant.

## Supplementary information

Supplementary info
