# Supplementary material for: Statins exacerbate glucose intolerance and hyperglycemia in a high sucrose fed rodent model
Source: Sci Rep. 2019 Jun 19;9:8825. doi: 10.1038/s41598-019-45369-8 (PMC6584635; doi:10.1038/s41598-019-45369-8)
Supplement: Supplementary file 1 — Supplementary info [file 41598_2019_45369_MOESM1_ESM.pdf]

Supplementary Figure

Statins exacerbate glucose intolerance and hyperglycemia in a high sucrose fed rodent model

Sriram Seshadri[1@], Naimisha Rapaka[2@], Bhumika Prajapati[1], Dipeeka Mandaliya[1], Sweta Patel[1], Christopher Shamir Muggalla[2], Bandish Kapadia[2#], Phanithi Prakash Babu [3], Parimal Misra[2]* and Uday Saxena[2]*

*Corresponding authors: *parimalm@drils.org; usaxena@drils.org*

Fig. S1:

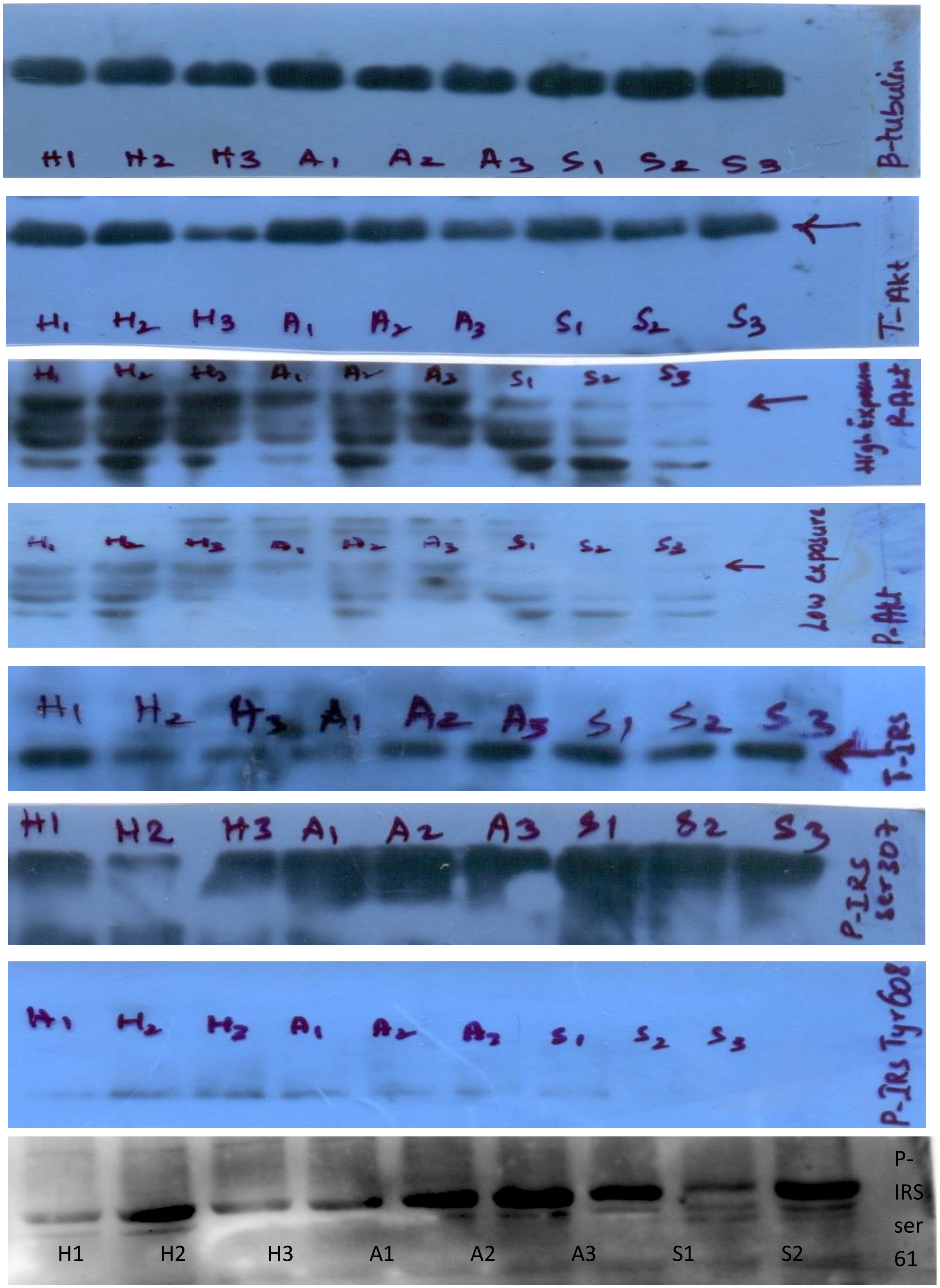
B-tubulin
H1 H2 H3 A1 A2 A3 S1 S2 S3
T-Akt
H1 H2 H3 A1 A2 A3 S1 S2 S3
High exposure P-Akt
Low exposure P-Akt
T-IRS
H1 H2 H3 A1 A2 A3 S1 S2 S3
P-IRS ser307
P-IRS Tyr608
H1 H2 H3 A1 A2 A3 S1 S2 S3
P-IRS ser 61
H1 H2 H3 A1 A2 A3 S1 S2

H1, H2 and H3: HSD rats;

A1, A2 and A3: HSD rats treated with Atorvastatin

S1, S2 and S3: HSD rats treated with Simvastatin

Fig. S1: Western blot raw data for β-tubulin, T-AKT, p-AKT, T- IRS1, p- IRS1 Ser^{307}, p-IRS1 Ser^{612} and p- IRS1Tyr ^{608} for rat muscle tissue of HSD rats ,atorvastatin and simvastatin treated rats. β-tubulin, T-Akt and p-Akt bands are from one gel whereas bands of T-IRS1, p- IRS1 ser ^{307}, p-IRS1 ser ^{612} and p-IRS1tyr ^{608} are from another gel.

Fig. S2A:

**Product Name : SIMVASTATIN USP**	**Item No. : 800000619**	Page No. : 2 of 2
Disp. Ref. No. : BF14005663	Manufacturing Date : July 2014	
Quantity : 104.70 kg	Retest Date : June 2017	
Batch No : BS14005634	A.R. No. : 40000008768	

USP, In-house & Customer's Specifications:

TESTS	OBSERVATIONS	LIMITS
*Assay: Content of Simvastatin (by HPLC)	99.3%	**Between 98.0% w/w and 101.0% w/w
$Residual solvents (by GC)	3.3 ppm<br>Below quantitation level<br>Below detection level<br>Below detection level<br>Below detection level	Methanol - NMT 100 ppm<br>Petroleum ether - NMT 50 ppm<br>Ethyl Acetate - NMT 1000 ppm<br>Tetrahydrofuran - NMT 100 ppm<br>Toluene - NMT 100 ppm
$Limit of Butylated Hydroxy Anisole (By HPLC)	86 ppm	Between 50 ppm and 150 ppm
#Loss on drying	0.12%	Not more than 0.30% w/w
#Particle size distribution	1.1 microns<br>3.2 microns<br>6.8 microns	d(0.1) - Less than 2.0 microns<br>d(0.5) - Less than 5.0 microns<br>d(0.9) - Less than 10.0 microns

REMARKS: The sample complies with the above tests as per USP, $In-house & #Customer's specifications
*On dried basis
**Inhouse limits

Storage : Preserve in well-closed containers, under Nitrogen at temperature below 30°C.

Fig. S2A: Purity data of simvastatin at the start of the study (Provided by manufacturer)

Fig. S2B:

SAMPLE INFORMATION

Sample Name:	ILS-SKJ-SM-ST	Acquired By:	System
A.R.Number:	CM19C036 (Simvastatin)	Sample Set Name:	25032019_004
Vial:	2	Acq. Method Set:	ILS_MET
Injection #:	1	Processing Method:	ILS PRO
Injection Volume:	5.00 ul	Channel Name:	230.0nm
Run Time:	35.0 Minutes	Proc. Chnl. Descr.:	PDA 230.0 nm
Date Acquired:	3/25/2019 8:23:41 PM IST		
Date Processed:	3/26/2019 9:54:31 AM IST		

Column: Eclips PLUS C18 250*4.6mm 5im (0.5mg Simvastatin dissolved in 1ml DMSO)
Mobile phase: A) 5MM AMMONIUM ACETATE B) ACN
T/%B: 0/10,23/90,30/90,31/10,35/10
Flow: 1.0ml/min, Diluent: ACN:WATER (80:20)

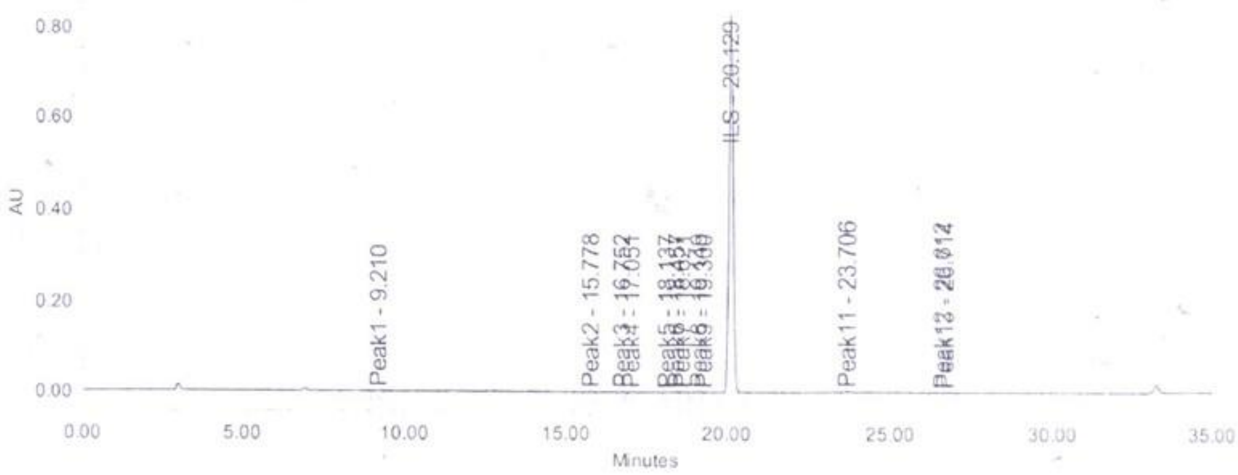

	Peak Name	RT	Height	Area	% Area	RT Ratio
1	Peak1	9.210	308	2418	0.03	0.46
2	Peak2	15.778	570	4782	0.06	0.78
3	Peak3	16.752	97	904	0.01	0.83
4	Peak4	17.051	166	1426	0.02	0.85
5	Peak5	18.137	57	457	0.01	0.90
6	Peak6	18.457	1214	10923	0.15	0.92
7	Peak7	18.621	592	4743	0.06	0.93

	Peak Name	RT	Height	Area	% Area	RT Ratio	
8	Peak8	19.149	1111	9666	0.13	0.95	
9	Peak9	19.300	94	969	0.01	0.96	
10	ILS	20.129	829546	7327999	99.30	1.00	->Simvastatin
11	Peak11	23.706	1058	10156	0.14	1.18	
12	Peak12	26.612	199	2433	0.03	1.32	
13	Peak13	26.714	192	2563	0.03	1.33	

Fig. S2B: Purity data of simvastatin generated in house using HPLC as described in the methodology section

Fig. S3A:

Product	: ATORVASTATIN CALCIUM (AMORPHOUS)	Customer name	: Dr.Reddy's Laboratories Ltd (FTO-3)
Batch No.	: ABHH001991	Date of Manufacture	: APRIL 2014
Batch Quantity	: 148.100 Kg.	Re-test Date	: MARCH 2017
A.R.No.	: 80000441473	Date Analysed	: 24.04.2014
Reference	: In-house / Customer	Specification No.	: S-08-AN-01/12

Storage : Preserve in tight containers at 25°C and protected from moisture.Excursions allowed between 15°C and 30°C. Always replenish with fresh silica gel and oxygen absorbent sachets whenever opened. Purge with Nitrogen and seal the triple laminated bag.

Packing condition : Fix the filter cloth in the packing machine. Material shall be kept in a clear white poly bag. Take out the white poly bag from the HDPE container. Fix the white bag to packing machine & run 3 cycles of Nitrogen purging & evacuation.Close the white poly bag with Nylon strap by making Nitrogen balloon in the bag and disturb the material by upside down the material. Fix the white poly bag to the packing machine.Run another 3 cycles of Nitrogen purging & evacuation.Close the white poly bag with Nylon strap by making Nitrogen balloon in the bag and disturb the material by upside down the material, tie with nylon strap. Fix the white poly bag to the packing machine. Run another 3 cycles of Nitrogen purging & evacuation. Close the white poly bag with Nylon strap by making Nitrogen balloon in the bag. Keep this white poly bag in black poly bag along with silica gel bags (2x50 gm) and Oxygen absorbent sachets (10000 ml capacity){i.e.10x1000 ml} capacity), run 6 cycles of Nitrogen purging and Evacuation in the black poly bag. Close the black polybag with Nylon strips by keeping Nitrogen balloon in the bag.Keep this in triple laminated bag along with silica gel bags (2x50 gm), run 3 cycles of Nitrogen purging and Evacuation in the triple laminated bag and seal it with heat sealer. Close the HDPE container lid.

Sl.No.	TEST	RESULT	SPECIFICATION
6.0	Assay by HPLC (On anhydrous basis)	100.2%w/w	Not less than 98.0% and Not more than 102.0%w/w
7.0	Residual solvents by GC		
7.1	**Method-I**		
7.1.1	Methanol	Less than LOQ.(LOQ=80ppm)	Not more than 1500ppm
7.1.2	Acetone	Not detected	Not more than 1500ppm
7.1.3	Isopropyl alcohol	Not detected	Not more than 1000ppm
7.1.4	Tertiary butyl alcohol	Not detected	Not more than 100ppm
7.1.5	Ethyl acetate	Less than LOQ.(LOQ=161ppm)	Not more than 5000ppm
7.1.6	Tetrahydrofuran	Not detected	Not more than 200ppm
7.1.7	Cyclohexane	Not detected	Not more than 1000ppm
7.1.8	Iso butyl formate	Not detected	Not more than 175ppm
7.1.9	Ethyl propionate	Not detected	Not more than 125ppm
7.1.10	n-Propyl acetate	Not detected	Not more than 200ppm
7.1.11	Ethyl isobutyrate	Not detected	Not more than 100ppm
7.1.12	Toluene	Not detected	Not more than 500ppm
7.1.13	Iso butyl acetate	Not detected	Not more than 200ppm
7.2	**Method-II**		
7.2.1	Dichloromethane	Not detected	Not more than 300ppm
7.2.2	Acetonitrile	Less than LOQ.(LOQ=60 ppm)	Not more than 410ppm
8.0	Specific optical rotation (On anhydrous basis)	-8.6°	Between -6.5° and -9.5°
9.0	Calcium content by AAS (On anhydrous basis)	3.5%w/w	Between 3.3% and 3.8%w/w
10.0	X-ray powder diffraction pattern	Matches with working standard.	To match with working standard
11.0	Impurity-E(Enantiomeric purity) by chiral HPLC (ent-Atorvastatin)	Less than LOQ.(LOQ=0.03%)	Not more than 0.15%
12.0	Nickel content by AAS	Not detected	Not more than 25ppm
13.0	Sodium content by AAS (On anhydrous basis)	1262 ppm	Not more than 4000ppm
*14.0	Particle size by Malvern		
14.1	10% of the particles	1µm	Not more than 2µm
14.2	50% of the particles	2µm	Not more than 5µm
14.3	90% of the particles	5µm	Not more than 15µm

Fig. S3A: Purity data of atorvastatin at the start of the study (Provided by manufacturer)

Fig. S3B:

SAMPLE INFORMATION

Sample Name:	ILS-SKJ-ATR-ST	Acquired By:	System
A.R.Number:	(Atorvastatin)	Sample Set Name:	29032019_003
Vial:	80	Acq. Method Set:	API RDL
Injection #:	1	Processing Method:	ILS PRO
Injection Volume:	5.00 ul	Channel Name:	245.0nm
Run Time:	35.0 Minutes	Proc. Chnl. Descr.:	PDA 245.0 nm
Date Acquired:	3/29/2019 9:14:50 PM IST		
Date Processed:	4/1/2019 10:52:20 AM IST		

(0.5mg of Atorvastatin dissolved in 1ml DMSO)

Column: X-BRIDGEC18 150*4.6mm 5µm
Mobile phase: A) 5MM AMMONIUM ACETATE B) ACN
T/%B:0/10,23/90,30/90,31/10,35/10
Flow: 1ml/min, Diluent: DMSO

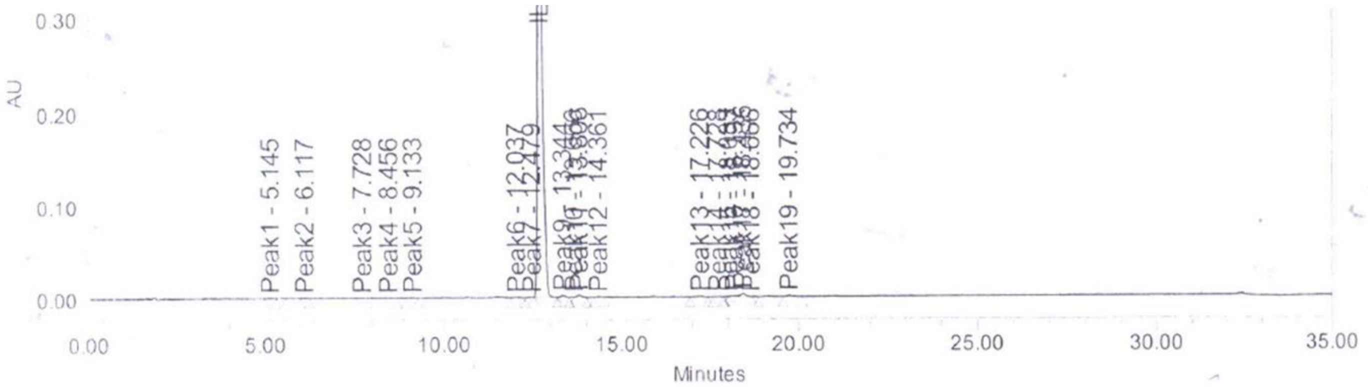

	Peak Name	RT	Height	Area	% Area	RT Ratio
1	Peak1	5.145	77	780	0.02	0.40
2	Peak2	6.117	150	1063	0.02	0.48
3	Peak3	7.728	83	882	0.02	0.61
4	Peak4	8.456	66	998	0.02	0.66
5	Peak5	9.133	285	2072	0.05	0.72
6	Peak6	12.037	257	2384	0.05	0.95
7	Peak7	12.479	245	2196	0.05	0.98

	Peak Name	RT	Height	Area	% Area	RT Ratio	
8	ILS	12.723	447051	4324346	97.11	1.00	-->Atorvastatin
9	Peak9	13.344	1962	15466	0.35	1.05	
10	Peak10	13.666	331	1230	0.03	1.07	
11	Peak11	13.806	2541	23247	0.52	1.09	
12	Peak12	14.361	642	5059	0.11	1.13	
13	Peak13	17.226	591	8490	0.19	1.35	
14	Peak14	17.728	182	1640	0.04	1.39	
15	Peak15	18.033	162	800	0.02	1.42	
16	Peak16	18.197	1190	12316	0.28	1.43	
17	Peak17	18.436	3336	35882	0.81	1.45	
18	Peak18	18.666	143	1034	0.02	1.47	
19	Peak19	19.734	1193	13261	0.30	1.55	

Fig. S3B: Purity data of atorvastatin generated in house using HPLC as described in the methodology section
